# Effect of quality control circle activities on the damage rate of endoscopic surgical instruments: A before-and-after study

**DOI:** 10.1097/MD.0000000000049524

**Published:** 2026-07-03

**Authors:** Ruiwen Zhan, JianWen Xu, Hong Qiang, Peihua Yuan, Huaqing Xu

**Affiliations:** a Department of CSSD, Shidong Hospital, Yangpu District, Shanghai, China; b Department of Nursing, Shidong Hospital, Yangpu District, Shanghai, China; c Operating Room, Shidong Hospital, Yangpu District, Shanghai, China; d Department of Equipment, Shidong Hospital, Yangpu District, Shanghai, China.

**Keywords:** endoscopic surgery instruments, quality control, quality control circle activity, reported loss rate

## Abstract

Endoscopic instruments are vulnerable to structural damage due to frequent reuse. This study aimed to evaluate the effectiveness of quality control circle (QCC) activities in reducing instrument damage. A multidisciplinary QCC team was established to implement interventions including enhanced staff training, standardized maintenance protocols, and peer-reviewed inspections. Instrument damage rates were compared before and after the intervention using chi-square analysis. The damage rate declined significantly from 2.33% to 0.27% (*P* <.001). Common issues such as missing parts and assembly confusion were markedly reduced, achieving a target improvement rate of 137.33%. Implementation of QCC activities in instrument management effectively reduced equipment damage, improved workflow, and enhanced patient safety. These findings suggest that QCC methods have potential for broader application in hospital quality-improvement initiatives.

## 1. Introduction

With the increasing number of minimally invasive surgeries each year, endoscopic surgical instruments have been widely used in various fields of minimally invasive surgery treatment and disease diagnosis.^[1]^ Our hospital is a second-grade Class A comprehensive hospital with 686 approved beds. In 2021, the number of endoscopic surgeries reached 1358, a 25.86% increase compared to the previous year. Our department processed approximately 11,297 endoscopic surgical instruments for cleaning and disinfection throughout the year. Endoscopic surgical instruments can be classified into 2 categories based on their structure: detachable and non-detachable. Their main characteristics include strong professionalism, diverse materials, complex structures, multiple joints and lumens, high precision, vulnerability, high cost, and a relatively short usage period.^[2–5]^

According to the 2017 guidelines from the Asia Pacific Society of Infection Control, surgical instruments should be regularly calibrated, and parts should be replaced according to the manufacturer’s instructions. Research indicates that the damage rate of endoscopic surgical instruments reaches 2.13%, necessitating timely detection and handling to avoid prolonging surgical time and reducing the success rate of surgeries, which could potentially endanger patients’ health. Therefore, ensuring the effective use of each endoscopic surgical instrument and reducing its wear rate are particularly crucial.^[6–9]^ However, conventional monitoring and staff training alone have proven insufficient in preventing repeated handling errors and equipment damage. Therefore, an innovative, systematic quality-improvement strategy is needed to address these persistent issues in surgical instrument reprocessing.

Quality control circles (QCC) originated from the statistics and management courses by Professor Deming and Professor Juran in the 1950s. In the 1990s, they were used for quality management in the medical and health fields in Taiwan. In 2001, QCC was introduced to medical institutions in China, aiming to improve the quality of medical service by raising the awareness of medical workers to spot and solve problems in their work.^[6]^

QCC refers to the people who share work in a certain field. They spontaneously work as a team to solve actual problems using quality control methods like Pareto and fishbone diagrams, in order to improve efficiency and personnel quality.^[5,10–12]^

Through this QCC activity, the sterilization supply center’s washing and disinfection processes and maintenance methods have been optimized. A quantitative evaluation scoring sheet for surgical instrument damage has been established, along with a peer review quality inspection and evaluation system integrated with the supply process. As a result, the damage rate of endoscopic surgical instruments has been reduced, extending their lifespan, reducing hospital property loss, and ensuring both medical quality and patient safety.^[13,14]^

Globally, QCC strategies have been adopted in various hospital settings to improve clinical performance and service quality. For instance, Wang et al highlighted the role of QCC in promoting sustained improvement in hospital-wide medical care through interdisciplinary collaboration.^[1]^ Chang et al demonstrated its application in enhancing patient transportation safety in emergency departments.^[2]^ These cases support the relevance of applying QCC in surgical instrument quality assurance, especially in high-risk environments involving reusable instruments. Furthermore, in surgical instrument management, Wang et al demonstrated that QCC interventions significantly reduced pretreatment failure rates, thereby enhancing standardization and staff adherence to reprocessing protocols.^[15]^

## 2. Methodology

### 2.1. Ethic statement

This study was conducted in accordance with the Declaration of Helsinki and was reported in accordance with the Strengthening the Reporting of Observational Studies in Epidemiology (STROBE) guidelines for observational studies. Ethical approval was obtained from the Institutional Review Board of Shidong Hospital, Yangpu District, Shanghai.

As the study involved only anonymized, retrospective quality-improvement data, the requirement for written informed consent was formally waived by the board. All data were collected from the hospital’s internal quality improvement and accreditation database in compliance with institutional and national regulations. The privacy of both patients and staff was strictly protected, and only validated data were used for analysis.

### 2.2. Formation of QCC

In August 2022, the Nursing Department organized the establishment of the “Together Circle” activity group, aimed at ensuring that the staff of the sterilization supply center carefully handle surgical instruments to provide safe, high-quality, and efficient services to the operating room, with departments cooperating with each other to collectively safeguard patients’ lives. The group consists of ten members, including 1 circle leader (the head nurse of the sterilization supply center), 1 mentor (Director of the Nursing Department), 1 team leader (head nurse of the operating room), and 6 members (including 1 doctor, 3 nurses, 1 equipment department clerk, and 1 infection control department clerk). Among the members of the QCC group, there are 2 deputy chief nurses, 2 deputy chief physicians, 1 engineer, 1 attending physician, 3 supervisory nurses, and 2 nurses. The circle leader is responsible for planning and tracking evaluations; the mentor mainly guides the progress; the team leader is responsible for data collection and technical support; and other members are assigned tasks according to their expertise. The group holds bi-monthly meetings during which members report progress, discuss issues, evaluate performance, and assess each stage of the activity.

### 2.3. Planning and implementation

The QCC group proposed 4 topics through brainstorming. Each topic was discussed and scored based on 5 aspects: superior policies, importance, urgency, feasibility, and the capabilities within the circle, using the “531” scoring method. Finally, based on the highest total score, “Reducing the damage rate of endoscopic surgical instruments” was determined as the theme for this period’s QCC activity. The formula for calculating the damage rate is: Endoscopic surgical instrument damage rate = (Number of instruments scrapped or damaged during the same period ÷ Total number of instruments used during the same period) × 100%.

**Figure 1.**
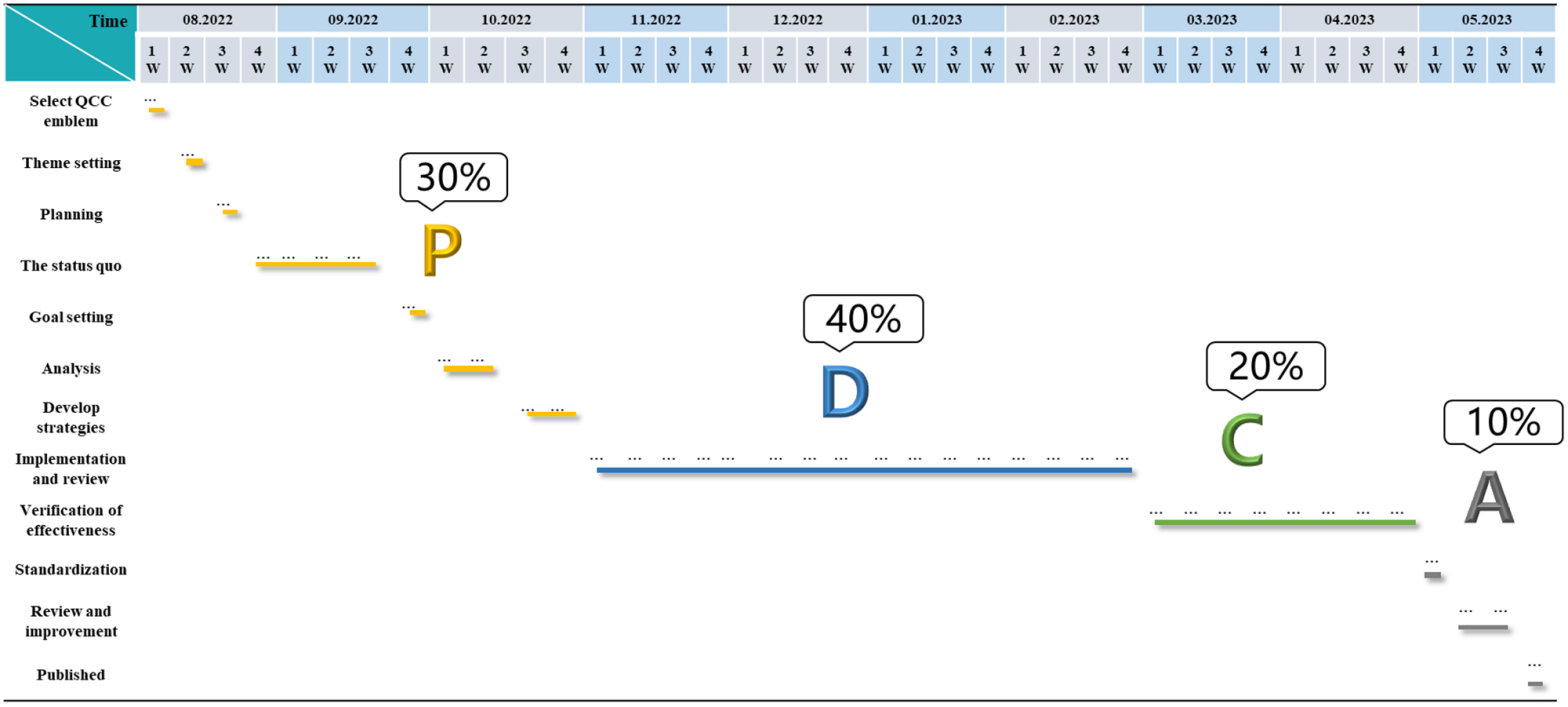
Gantt chart of the QCC activity plan based on the PDCA cycle. The team developed a detailed activity schedule following the PDCA framework. Responsibilities were assigned, and implementation steps were evaluated to ensure structured execution. QCC = quality control circle, PDCA = plan-do-check-act.

After determining the activity theme, a Gantt chart was created (Fig. 1), and a 10-month activity plan was devised based on the plan-do-check-action (PDCA) cycle, with a time ratio of 3:4:2:1.

### 2.4. Confirmation of effectiveness

**Figure 2.**
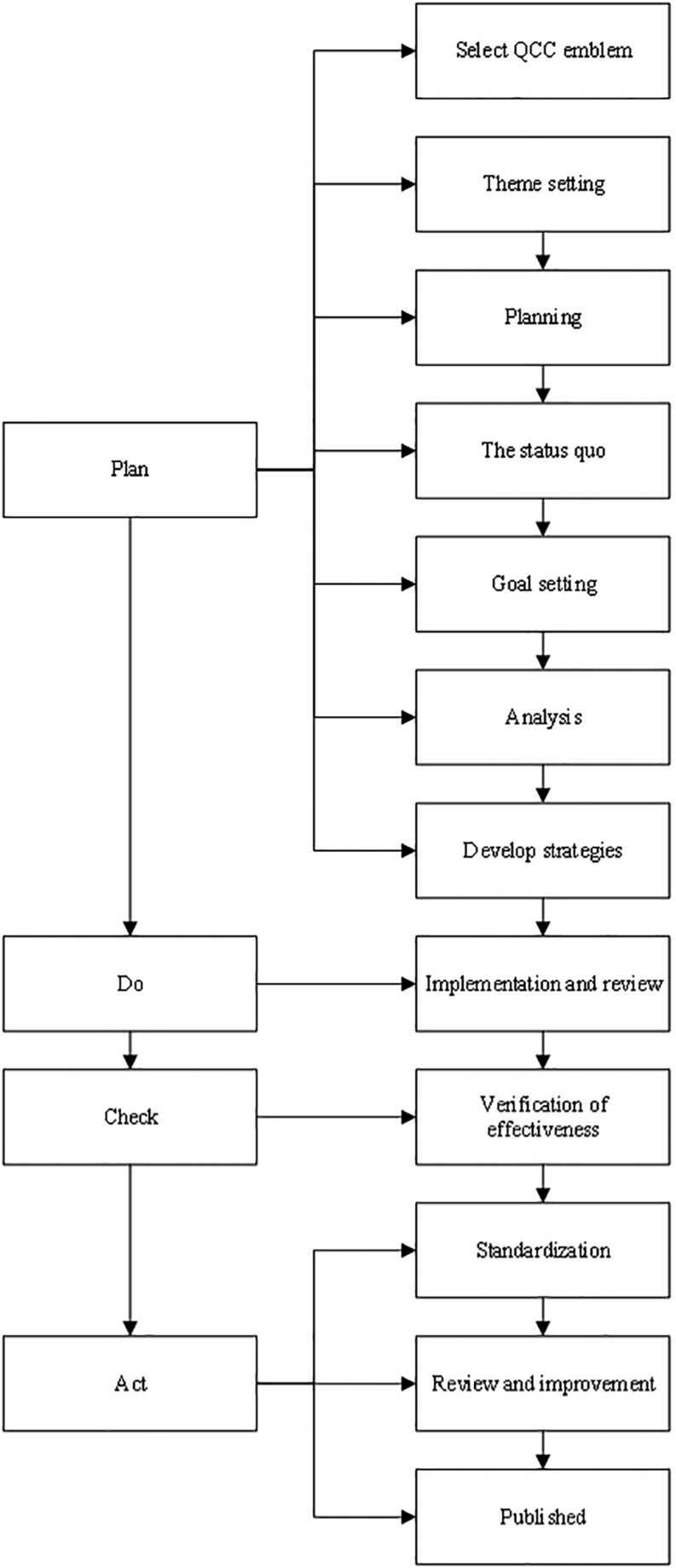
Protocol of the quality control circle. The basic steps of QCC activity generally followed the Deming cycle (PDCA cycle). The 4 stages (PDCA) were realized through 10 basic steps. PDCA = plan, do, check, and act, QCC = quality control circle.

The duration of this QCC activity was set at 10 months (from August 2022 to May 2023). The basic steps of QCC activity generally followed the Deming cycle (PDCA cycle). The 4 stages (PDCA) were realized through 12 basic steps (Fig. 2). The effect, including tangible and intangible results, was evaluated after the implementation of the strategies. The tangible results included achievement rate and improvement rate. The intangible results were presented by multiple indicators in a radar map.

### 2.5. Investigation of the status quo and goal setting

**Figure 3.**
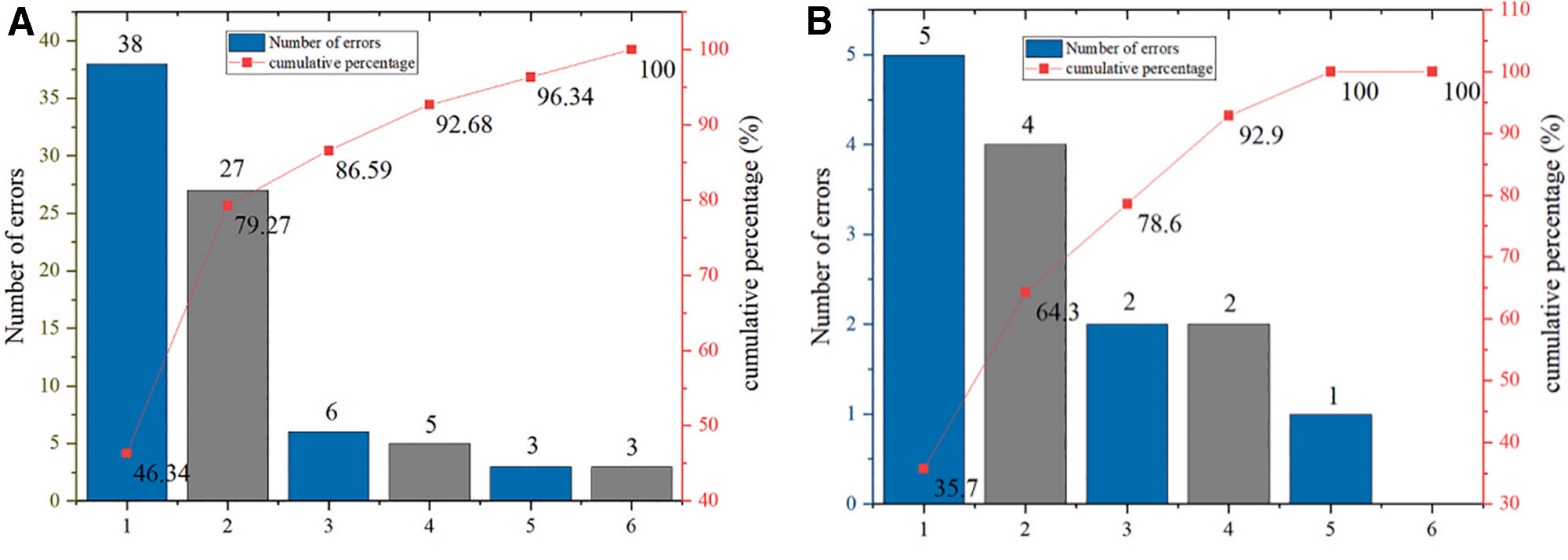
Pareto analysis (A1B1. missing instrument components; A2B2. confusion between inner core, outer sheath, and handle; A3B3. Loose screws on instruments; A4B6. Deformation of instrument tips; A5B5. Axis joint not operating smoothly; A6B4. Blunt scissors).

During the period from August to October 2022, the disinfection supply center processed a total of 3520 endoscopic surgical instruments. Through the use of a self-made checklist, circle members discovered a total of 82 damaged instruments, including 38 with missing components, 27 with internal cores, outer sheaths, and handles confused, 6 with loose or fallen screws, 5 with deformed tips, 3 with rough joint articulation, and 3 with dull scissors. The damage rate was calculated at 2.33%. The criteria for determining damage were: instruments exhibiting breakage or missing parts, confusion rendering assembly impossible, performance impairment, or posing safety hazards, and being irreparable after maintenance. Based on the analysis results of the Pareto Principle (80/20 rule) as shown in Figure 3, the cumulative percentage of missing components and internal core, outer sheath, and handle confusion was 79.27%, thus identifying these 2 as the focal points for improvement in this activity.

Based on the members’ situation, the calculation formula shows that the circle’s capability is 81.44%. Through investigation, the current rate of damage to endoscopic surgical instruments before improvement is 2.33%, with a focus on improvement of 79.27%. According to the formula, the target value is derived by subtracting the current rate from the product of the current rate, improvement focus, and circle capability. The calculated target value after improvement is 0.83%, with an improvement rate of 64.38%.

**Figure 4.**
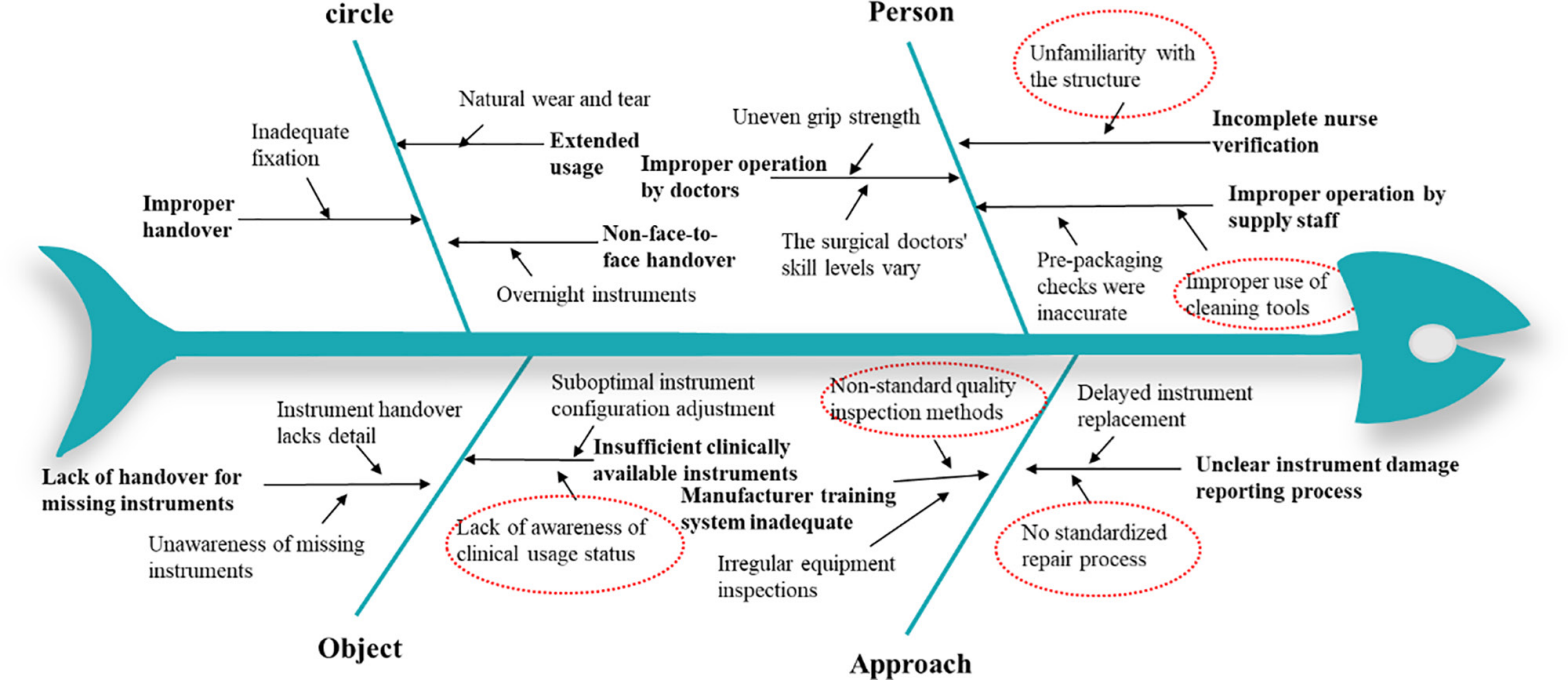
Fishbone (Ishikawa) diagram of root causes for missing components in endoscopic surgical instruments. The diagram outlines potential contributing factors categorized into 5 domains: personnel, methods, equipment, materials, and environment.

**Figure 5.**
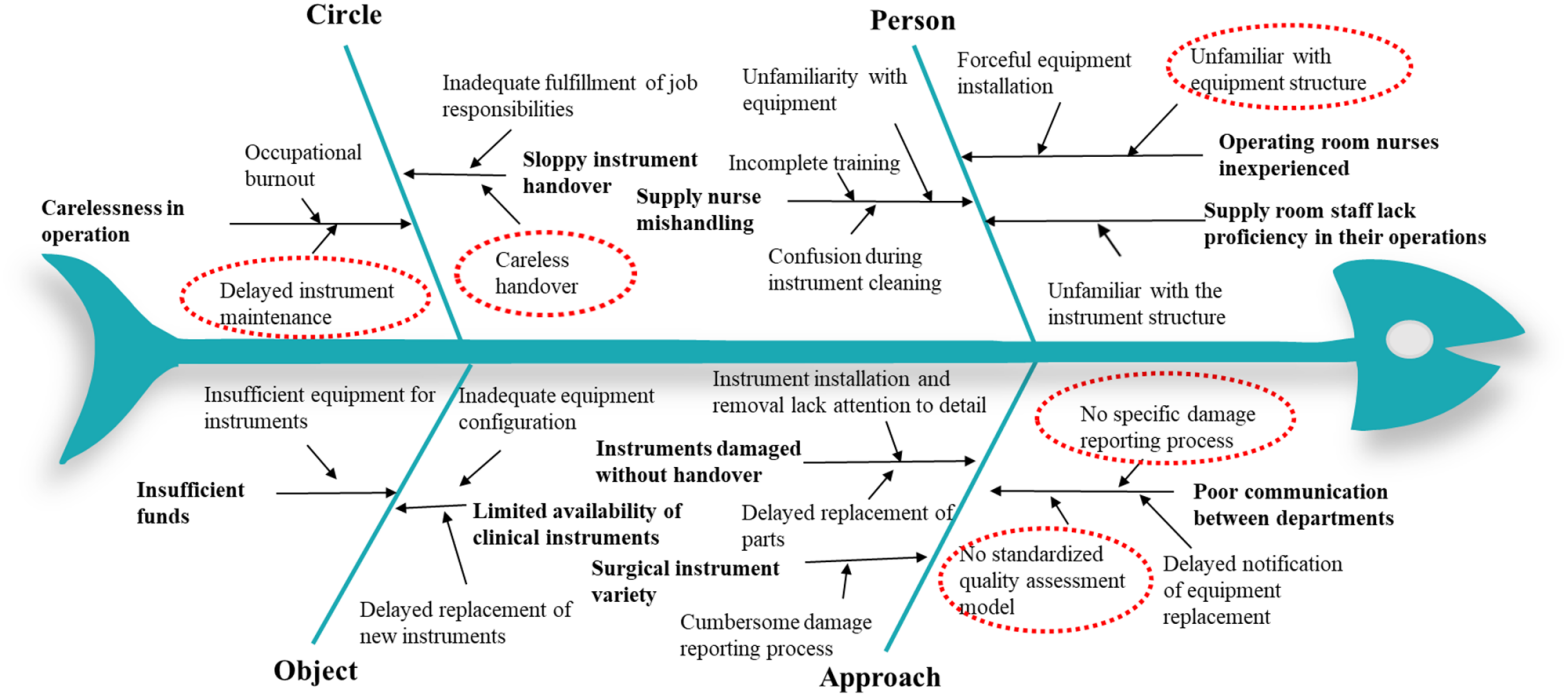
Fishbone (Ishikawa) diagram of root causes for assembly errors between inner cores and outer sheath handles in endoscopic instruments. The diagram illustrates the main contributing factors responsible for instrument mismatches during reassembly, categorized by domain.

The team members created a fishbone diagram from aspects like people, machines, methods, environment, knowledge, trust, and behavior, analyzing 2 key factors: the absence of instrument components (Fig. 4) and confusion between the inner core, outer sheath, and handle (Fig. 5). Altogether, they identified 46 reasons. Each reason was rated for its importance, with scores of 5 for extremely important, 3 for important, and 1 for unimportant. Utilizing the Pareto Principle (80/20 rule), they identified 9 key factors, including nurses’ unfamiliarity with the structure of laparoscopic instruments, non-standardized quality inspection methods, lack of utilization of relevant equipment for quality checks, absence of a unified evaluation system for instrument loss, nonstandard guidance of auxiliary personnel by nurses, incorrect use of cleaning brushes, inadequate specialized instrument training, insufficient supervision and guidance, and the lack of a unified quality assessment mode for instrument supply. Employing the Pareto Principle once again, they verified the true causes using a self-made checklist, ultimately confirming 4 genuine reasons, namely nurses’ lack of familiarity with the structure of laparoscopic instruments, improper use of cleaning tools by auxiliary personnel, nonstandard quality inspection methods, and the absence of a unified quality assessment mode for instrument supply.

### 2.6. Strategies and implementations

All circle members discussed and formulated 4 groups of strategies based on the 4 root causes, totaling 7 strategies. Chaired by the circle leader, members rated each strategy based on feasibility, autonomy, and effectiveness, with scores of excellent (5 points), good (3 points), and poor (1 point). The maximum total score for each strategy was 150 points. According to the 80/20 principle, strategies scoring above 120 points were deemed implementable. Following comprehensive evaluation, the following 4 strategies were determined.

The team developed a training improvement plan using proactive techniques, including automatic prompts, visual warnings, color-coded labels, and classification systems to prevent workflow errors and other technical means before errors occur or during operation. This aims to prevent operators from becoming careless or making mistakes in the workflow, thereby avoiding errors or adverse events. Applying the principle of conformity to classify and label the cleaning of endoscopic instruments, ensuring that the choice of cleaning brushes corresponds to the lumens and creating diagrams to prevent mishandling that may damage the instruments. Employing the “press once, loosen twice, pull 3 times” sequential principle to train staff in the correct disassembly and assembly of endoscopic instruments. Utilizing the principle of hierarchy to train staff in inspecting and maintaining packaged instruments, ensuring that each instrument’s shaft, screws, buttons, caps, gaskets, etc, are checked 1 by 1 and marked accordingly according to the instrument checklist.^[6]^

Based on the manufacturer’s instructions, a unified rating system for evaluating equipment damage is established. The rating consists of subjective and objective components. Subjective ratings are based on 3 scrap indicators outlined in the “Management Measures for Medical Equipment in Medical and Health Institutions”: endangering personal safety and health, malfunction or low functionality, outdated technology, and excessively high maintenance costs, covering 3 aspects: equipment safety, usability, and cost-effectiveness. These aspects are further subdivided into 8 specific evaluation criteria, using the Likert 5-level scoring method: equipment safety (integrity of appearance, degree of corrosion, frequency of maintenance, risk of adverse events), usability (equipment performance, level of technological advancement), and cost-effectiveness (economic benefits, maintenance costs), totaling 40 points. Objective ratings are mainly based on the manufacturer’s instructions and are calculated by deducting points based on the designed service life or usage frequency. Scrap items include severe damage beyond repair and mandatory failed inspections. The equipment damage score is calculated as the average of subjective ratings from team members plus 60% of the objective score. If the score is 80 or higher, scrapping is recommended; if it involves direct scrapping items, they are scrapped directly.^[2–5]^

The inspection of laparoscopic surgical instruments includes optical lenses, light guides, instruments, and accessories. This encompasses the correct disassembly and assembly processes, as well as standardized inspection and testing, aimed at assessing the functional status and cleaning quality of the instruments, effectively achieving maintenance and upkeep operations. We referenced the inspection and packaging procedures outlined in the “2018 Guidelines for Cleaning, Disinfection, and Sterilization of Rigid Endoscopes” to refine the inspection and maintenance operations of optical lenses, light guides, and laparoscopic instruments. We used an automatic rigid endoscope detector to test the 18 optical lenses and 21 light guides currently in circulation, including lens transmittance, illumination (FB), depth of field (FC), color correction, and field of view angle. The test results showed that the average light transmission of 3 optical lenses had decreased, indicating a need for repair or replacement, while the remaining 15 lenses were in normal condition. This process ensures that operators inspect each axis and groove to avoid stiffness during assembly and use by surgeons, effectively reducing the risk of instruments being subjected to violent disassembly.^[8]^

The team revised the quality inspection form related to equipment handover, transportation, cleaning, and sterilization. Indicators were categorized into primary (knowledge, belief, behavior) and secondary components (e.g., understanding structure, operating procedure compliance, use of appropriate brushes, etc). Primary indicators include knowledge, belief, and behavior; secondary indicators include understanding of the equipment, understanding of the equipment structure, understanding of equipment disassembly and assembly, adherence to operating procedures, reasonableness of procedures, face-to-face handover checks, correct disassembly and assembly, adherence to manufacturer’s instructions for selecting cleaning and disinfection methods, safe transportation, effective use, and other 14 inspection items. These items are supervised by circle members, and 2 people are responsible for counting and verifying errors.^[11]^

In addition, the observation indicators include comparing the pre- and post-implementation of the quality management circle activities in terms of the rate of damage to endoscopic surgical instruments, as well as comparing the scores of circle members in 6 aspects of quality management circle methods, teamwork, communication skills, responsibility, professional skills, and problem-solving skills, ranging from 1 to 5 points.

### 2.7. Statistical processing

We analyzed the data before and after improvement using SPSS 22.0 statistical software. Count data were presented as percentages and analyzed using the χ^2^ test, with differences considered statistically significant at *P* <.05. No missing data were identified in the aggregate quality-improvement database used for the pre- and post-intervention comparison.

## 3. Results

### 3.1. Tangible results

Before improvement, the damage rate of endoscopic surgical instruments was 2.33%, with a target value of 0.83%. After improvement, it dropped to 0.27%. The target achievement rate reached 137.33%. Comparing the damage situation of instruments before and after improvement, from August to October 2022, there were a total of 3520 instruments, with a damage rate of 2.33%. From November 2022 to February 2023, the damage rate of 5213 instruments decreased to 0.27%. This improvement shows significant statistical differences (*P* <.001). Details of the distribution of endoscopic instrument damage types before and after QCC implementation are presented in Table 1.

**Table 1:** Distribution of endoscopic instrument damage types pre- and post-QCC implementation.

Type of damage	Improved (N = 5213)		Before improvement (N = 3520)		χ^2^	*Ρ*
	Number of damages (N)	Damage rate (%)	Number of damages (N)	Damage rate (%)		
Loss of instrument components	5	0.10	38	1.08	41.493	<.001
Inner core, outer sheath, handle confusion	4	0.08	27	0.77	28.308	<.001
Loose screws	2	0.04	6	0.17	4.006	>.05
Deformed tip	0	0	5	0.14	7.409	>.05
Stiff joint axis	1	0.02	3	0.09	2.002	>.05
Blunt scissors	2	0.04	3	0.09	0.806	>.05
Total	14	0.27	82	2.33	82.094	<.001

QCC = quality control circle.

Before the improvement, there were 3520 units of endoscopic surgical instruments, involving 8 indicators in the processing workflow, including the handover of instruments between the operating room and the disinfection supply center, the disassembly and assembly of instruments by personnel in the disinfection supply center, knowledge of the instruments, selection of cleaning brush models, instrument maintenance methods, disassembly and assembly of instruments by operating room personnel, understanding of instruments, and the manner in which surgeons use and handle instruments. After the improvement, the number of endoscopic surgical instruments increased to 5213 units. We compared these 2 sets of data, calculated the number of errors, and calculated their percentages. The results indicate that the differences between these data sets are statistically significant (*P* < .001). The comparison of error frequencies in the handling of endoscopic surgical instruments before and after QCC intervention is shown in Table 2.

**Table 2:** Comparison of error frequencies in the handling of endoscopic surgical instruments before and after QCC intervention.

Type of issues	Improved (N = 5213)		Before improvement (N = 3520)		χ^2^	*Ρ*
	Number of errors (N)	Error rate (%)	Number of errors (N)	Error rate (%)		
Instrument handover between OR and disinfection supply center	2	0.04	21	0.60	24.927	<.001
Staff disassembling instruments	1	0.02	25	0.71	33.804	<.001
Surgical staff handling instruments	1	0.02	18	0.51	23.446	<.001
Sterilization center staff understanding of instruments	2	0.04	31	0.88	39.601	<.001
Operating room staff understanding of instruments	1	0.02	28	0.79	38.256	<.001
Disinfection supply center staff selecting brush models	0	0	20	0.57	29.687	<.001
Disinfection supply center staff maintenance methods	0	0	45	1.28	66.989	<.001
Surgeons’ use of surgical instruments	1	0.02	25	0.71	33.804	<.001
Total	8	0.10	213	6.05	296.30	<.001

QCC = quality control circle.

### 3.2. Intangible results

**Figure 6.**
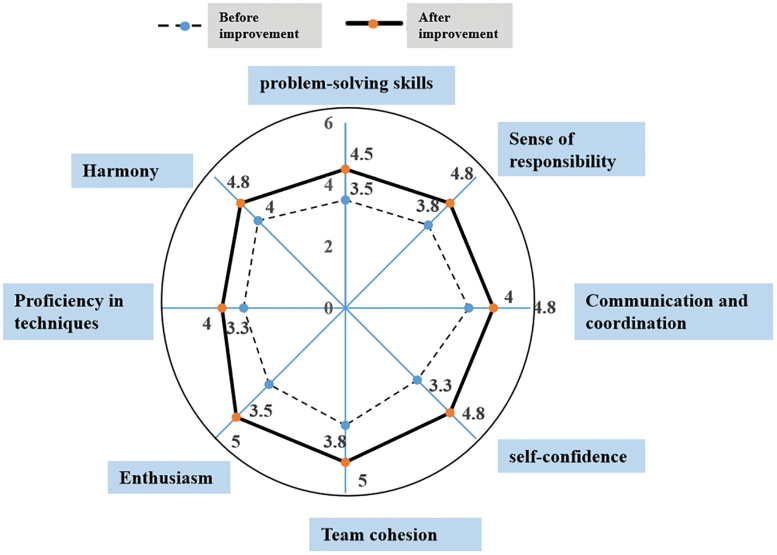
Radar chart of intangible outcomes related to QCC intervention. The chart illustrates changes in team collaboration, communication efficiency, staff satisfaction, and awareness of quality control before and after QCC implementation. QCC = quality control circle.

The radar chart (Fig. 6) demonstrates a significant improvement in 6 aspects, including quality management techniques, teamwork, communication, sense of responsibility, professional competence, and problem-solving ability among the circle members during this practical activity.

## 4. Discussion

During the QCC intervention, the damage rate of laparoscopic surgical instruments was significantly reduced from 2.33% to 0.27% (*P* < .001), surpassing the target value of 0.83%. Nonetheless, this provided a basis for improving and optimizing the management of laparoscopic surgical instruments. We employed a combination of qualitative and quantitative methods to conduct a thorough analysis of the damage situation of surgical instruments, effectively reducing the damage and scrappage of laparoscopic surgical instruments. By strengthening the inspection, maintenance, and upkeep of these instruments, we enhanced their lifespan, thereby reducing the rate of damage.^[2,4]^

The QCC activity played a regulatory and supervisory role in the management of laparoscopic surgical instruments. Through interdisciplinary brainstorming sessions, it broke the inertia thinking pattern regarding the damage of laparoscopic surgical instruments and formulated corresponding measures for 4 root causes: establishing standard operating procedures for the lifecycle of laparoscopic surgical instruments, implementing peer quality inspections, and developing a scoring system for instrument damage evaluation.^[5,6]^ We proactively identified issues in the workflow and effectively reduced the damage rate caused by missing instrument components and confusion between internal and external sheaths of the instrument handle. Moreover, it prevented mutual blame between the disinfection supply center and the operating room.^[8,11]^ The support staff at the disinfection supply center could select the correct cleaning brushes based on their labels and perform operations according to the “four-point” maintenance schematic of the instruments, thus preventing errors. The error rates for cleaning brush selection and instrument maintenance methods decreased from 0.57% and 1.28%, respectively, before improvement to zero afterward. We provided operational standards for staff with zero experience, eliminating the need for repetitive theoretical instructions from management, thereby facilitating smoother communication and handover between the operating room and the disinfection supply center. By combining manufacturer instructions and recommendations for disinfection parameters, circle members developed the “Yangpu District East Hospital Instrument Damage Assessment Form,” starting from scratch through practical implementation, and achieved quantified scoring based on subjective and objective evaluation indicators. This initiative established executable and scalable standards and directions for the safe use and quality management of reusable instruments, aiding in the timely detection and resolution of issues and risks in instrument management.^[5]^

Through cross-departmental collaboration and the application of QCC activities, effective management of endoscopic surgical instruments has been achieved, significantly reducing economic and resource wastage caused by instrument damage. Simultaneously, this initiative has facilitated communication between the sterilization supply center and clinical departments, enhancing professional competence and ethical standards, bolstering awareness of medical quality and safety, and ensuring the provision of safer and more reliable medical services to patients. Furthermore, the increased utilization of surgical instruments by doctors and the reduction of medical costs are of significant importance.^[13]^

Through studying the impact of QCC activities on reducing the rate of damage to endoscopic surgical instruments, we have revealed its significant role in improving the effective utilization of these instruments and reducing medical costs. However, we have also identified some shortcomings. Future research could explore areas such as expanding the sample size and optimizing the application of QCC activities. We believe that this study can provide strong evidence and guidance for the management of endoscopic surgical instruments, further enhancing the level of management and providing safer and higher-quality medical services for patients.^[14]^

## 5. Conclusion

This study demonstrated that implementing QCC activities significantly reduces the damage rate of endoscopic surgical instruments, decreasing from 2.33% to 0.27%. The intervention successfully optimized cross-departmental collaboration, standardized instrument management protocols, and improved personnel training and inspection processes. These outcomes not only enhance patient safety but also improve institutional efficiency and cost-effectiveness. Future studies should explore the long-term sustainability of QCC interventions and their scalability across diverse clinical settings.

## Author contributions

**Conceptualization:** Ruiwen Zhan, Huaqing Xu.

**Data curation:** Ruiwen Zhan, Huaqing Xu.

**Formal analysis:** Ruiwen Zhan, Huaqing Xu.

**Funding acquisition:** Ruiwen Zhan, Hong Qiang, Huaqing Xu.

**Investigation:** Ruiwen Zhan, Huaqing Xu.

**Methodology:** Ruiwen Zhan, Hong Qiang, Huaqing Xu.

**Project administration:** Ruiwen Zhan, Huaqing Xu.

**Resources:** Ruiwen Zhan, JianWen Xu, Huaqing Xu.

**Software:** Ruiwen Zhan, JianWen Xu, Peihua Yuan, Huaqing Xu.

**Supervision:** Ruiwen Zhan, JianWen Xu, Peihua Yuan, Huaqing Xu.

**Validation:** Ruiwen Zhan, JianWen Xu, Huaqing Xu.

**Visualization:** Ruiwen Zhan, Huaqing Xu.

**Writing – original draft:** Ruiwen Zhan, Huaqing Xu.

**Writing – review & editing:** Ruiwen Zhan, Huaqing Xu.
